# Memoires de l'Académie Royale de Medecine. Tome XI

**Published:** 1845-07

**Authors:** 


### Memoires de l'Académie Royale de Medicine. Tome XI. 4to. pp. 800. Paris, 1845.

Although the present volume of the French Academy Memoirs is inferior in interest to some of its predecessors, and contrasts disadvantageously in practical value with our Medico-Chirurgical Transactions, yet it contains much well worthy of the attention of our readers. There is the same fault in it that characterises almost every work issuing from the French medical press—inordinate diffuseness. Paper and print had indeed need be cheaper on the Continent than with us, but, unless a book is to be considered valuable in proportion to the number of words it contains, it is an unfortunate circumstance for the reader that they are so. Casting aside the endless repetitions, the circumlocutory verbosity, and the irrevalent observations, which disfigure them, there can be no doubt that both author and reader would gain vastly by the compression of any of these works into one-third of their compass. Certainly the present volume would gain by the process.

Passing over the eulogia upon the deceased members of the Academy, and a comparative view of the recent progress of Medicine and Surgery in France, we come to the consideration of the various Cases and Memoirs.

### A MEMOIR ON URETRO-PLASTIE. By M. *Segalas*.

M. Segalas, in a letter addressed to M. Dieffenbach in 1840, stated his opinion, that the attempts at reparation of a breach of substance of the urethra had hitherto failed in consequence of the contact of the urine with the parts concerned not having been prevented; and, at the same time, detailed the particulars of a case in which, this latter object being attained by making a preliminary incision in the perineum, and introducing a catheter through it into the bladder, a complete cure resulted. A second case was similarly treated by M. Ricord, and a third forms the subject of the present communication.

This patient, aged 30, exhibited a large loss of the spongy portion of the urethra, the consequence of gangrene supervening upon a ligature he had attached to the penis when a child. The urine and seminal fluid passed entirely by this aperture, which was an inch in length, and occupied the entire thickness of the urethra, a deep transverse cicatrix being observed also to surround the corpus cavernosum. The portion of the urethra anterior to the loss of substance was much narrowed, but easily admitted a probe, and when the patient approached the two orifices of the defective portion he could force urine through it. The operation was divided into three stages; in the first, the prepuce, which was phymotic, was divided, in order to relax the parts afterwards to be operated upon. Six days after, a free opening was made into the membranous portion of the urethra, and a gum-elastic catheter introduced. The urine thus being prevented coming in contact with the canal, and the parts being in a tranquil state, the operation was completed, three weeks after the first incision of the prepuce, by dividing the edges of the defective portions of urethra and bringing them into contact over a bougie by means of the twisted suture. The wound healed kindly, except at one minute point, which remained fistulous for a very long period, notwithstanding the application of various cauteries, &c. It however eventually completely healed, though not until its orifice had been incised. The operation was performed the 18th August, and the catheter removed from the perineum the 26th November. Dieffenbach had objected to this operation, that the wound made in the perineum might itself remain fistulous. Such has not been the case in the other cases reported, and in the present one, it closed in a few days after the removal of the instrument. Although perfectly able to evacuate the bladder by the urethra, the patient was recommended to do so for a considerable time by means of a catheter. Throughout the treatment of the case, neither fever or irritation manifested itself.

### On Meningeal Apoplexy. By Dr. *Prus.*

Dr. Prus enters, in this memoir, into a comparison of the two different forms of this affection, that in which the hæmorrhage takes place into the cavity of the arachnoid, and that in which the blood is effused between the arachnoid and the pia-mater. His position as physician to the Salpêtrière, where so many aged persons are admitted, has afforded him more opportunities of observing these cases than fall to the lot of most practitioners. He observes:—

"Affections of the serous membranes play a great part in the pathology of old age. It would seem that the diminution of cutaneous perspiration determines in the serous membranes, as well as the pulmonary mucous membrane, a more abundant exhalation, and frequently also an afflux of blood which predisposes them to phlegmasiæ and hæmorrhages. These phlegmasiæ should the more attract the attention of the practitioner, inasmuch as they often progress in a latent manner, and may become fatal in a very few days. This observation applies especially to the pericarditis and meningitis of the aged. In a forthcoming memoir I shall occupy myself with this subject, as important as it is little known. Hæmorrhage from serous membranes, although less frequent than their inflammation, is yet by no means rare. I have observed it in the peritoneum, the pleura, the pericardium, in the cerebral and spinal arachnoid, in the cerebral ventricles, and in the infra-arachnoid, cavity. The hæmorrhage into the arachnoid cavity, and into the vascular tissue of the pia mater, known by the name of *meningeal apoplexy*, is that which will engage my attention to day."

Although this affection has obtained its present name only since 1819, when Serres distinguished it from hæmorrhage into the cerebral parenchyma, yet Morgagni has left us observations of almost all its varieties. Since Serres wrote, numerous practitioners, as Rostan, Blandin, Abercrombie, Andral, &c. &c. have published interesting cases. M. Boudet, in 1840, produced an important monograph upon the subject, in which he collected 41 cases, observed by himself and preceding writers. He has completely proved that the coagula found in the parietal arachnoid are not, as stated by many writers, placed between the arachnoid and dura-mater. Their enclosure in a pseudo-membrane has given rise to the error. Dr. Prus details the particulars of 16 cases, all but one being corroborated by careful post-mortem inspection. In six of these the hæmorrhage was seated between the arachnoid and pia mater. In nine others blood was found, either in a fluid or coagulated state, in the cavity of the arachnoid itself.

In the *sub-arachnoid variety* the blood is found, either fluid or coagulated, on any part of the surface of the brain or spinal marrow, being separated from the nervous substance by the pia mater only, which is even sometimes penetrated. When the effusion is very considerable it is found at the base, and is generally due to the rupture of an artery. Only three cases of rupture of the sinus, recorded by MM. Serres, Douglas and Felassier, are known. It is this form of the disease alone which results from the rupture of an artery or considerable vein, although it may, as the other form, be also produced by exhalation. However long the effusion may have existed prior to death, it has never been observed, as in the other variety, to become enveloped in a false membrane. If the name *apoplexy*

were confined to cases in which paralysis of motion and sensibility existed, these cases could hardly be so termed ; for, of twelve recorded, as resulting from rupture of cerebral arteries, in two only hemiplegia existed. Coma has been the only symptom constantly observed by the author. No cyst, indicative of absorbed effusions, has ever been detected.

"The term *sudden apoplexy,* which can perhaps never be justly applied to hæmorrhage into the cerebral pulp, or even into the cerebellum and protuberance, is admissible on the other hand, in a certain number of sub-arachnoidean hæmorrhages. Cerebral congestion, or *coup-de-sang,* serous apoplexy in the ventricles and sub-arachnoid cavity, hæmorrhage into the subarachnoid cavity and ventricles, alone merit, and that only in certain cases, the name of sudden apoplexy.'

Dr. Prus observes that, it might seem at first sight that an effusion of blood would produce the same effects, whether occurring within the cavity of the arachnoid, or between this membrane and the pia mater ; but, in point of fact, two diseases result, differing in their anatomical characters, symptoms, progress, termination, and treatment. The following are some of his conclusions upon this point.

"*Anatomical Characters.*—In one-half the cases at least, hæmorrhages between the arachnoid and pia-mater arise from arterial or venous rupture, while in the intra-arachnoid apoplexy the blood is never effused but by exhalation. In the sub-arachnoid form the blood mingles with the cephalo-spinal fluid of the ventricles and spine, to which it may convey irritating properties, as well as discoloration. In the other form it has no communication with this fluid. Blood effused beneath the arachnoid never becomes covered with a false membrane, while that contained within its cavity always does, if it have remained for four or five days. In the sub-arachnoid form, even where the hæmorrhage has been intermitting, sufficiently distinct differences in appearance and consistence of the coagula do not exist to enable a difference in their age to be determined. This, in many cases of intra-arachnoid hæmorrhage, can be done. So in the former case the effused blood does not become adherent to the walls of the membranes, which it does in the latter. Observers have met with cysts in the cavity of the arachnoid containing blood or traces of blood, and which they have regarded as processes of cure; but these have never been discovered in the other form.

"*Symptoms.*—In sub-arachnoid apoplexy paralysis is a very rare occurrence, it having been observed only three times in twelve cases of rupture of arteries, and never in those in which the blood resulted from rupture of the sinuses, or from exhalation. In six out of eight cases of sub-arachnoid apoplexy there was paralysis of motion. That of sensation is much more rare. In none of the six cases of sub-arachnoid apoplexy was there any sudden loss of consciousness, and in three instances out of eight of the intra-arachnoid form only did this occur. Somnolence and coma have shewn themselves nearly constantly towards the termination of both diseases: but, in the sub-arachnoid apoplexy, these symptoms are usually only preceded or accompanied by uneasiness, feebleness, redness and heat of the face and head; while, in the intra-arachnoid apoplexy, there is almost always observed cephalalgia, dry tongue, fever and delirium—symptoms due to the presence of inflammatory action.

"It is a remark well worthy of the attention of the practitioner that blood effused, even in large quantities, beneath the arachnoid, may diminish the intellectual powers, but it does not pervert them; *i. e.* it does not cause delirium; while, on the other hand, it is rare for blood to be effused for more than four days in the cavity of the arachnoid without inducing it. It is about this time that the coagulum becomes covered with a false membrane."

The essential symptoms of apoplexy of the cerebral pulp, sudden loss of consciousness, and the production of paralysis, are thus not always present in the meningeal form. In fourteen cases the deviation of the mouth, so common in ordinary apoplexy, has been observed but once. The persistence and intensity of the somnolence and coma constitute a principal characteristic of the meningeal form. Sub-arachnoid apoplexy has not been known to continue longer than eight days; but the duration of the intra-arachnoid form may be a month or more; or, as cysts found long after the period of effusion prove, it may now and then be cured. To this end the author recommends vigorous and sustained antiphlogistic treatment in this form of the disease.

### On Œdema of the Glottis. By *F. Valleix.*

The Academy of Medicine proposed the following subject for their prize. *State the causes of œdema of the glottis, describe its progress, successive symptoms, and differential diagnosis. Discuss the advantages and inconveniences of tracheotomy in its treatment.*

Prior to the proposition of this question the attention of M. Valleix had been drawn to the subject during the composition of his much esteemed *Guide du Medecin Pratique.* Although œdema of the glottis was described by Bayle in 1808, its right of reception into our nosologies as a distinct affection has been frequently contested; and certainly of the forty recorded cases collected by M. Valleix for the present essay, there are several to which this designation can be ill-applied. Indeed, as will be shortly seen, the description of our author embraces all inflammatory affections of the upper orifice of this tube capable by their resulting depositions of obstructing the admission of air. Practically this is of little consequence, since the same principles of treatment are applicable. He adds three cases from his own practice, and refers to two occurring in children, as reported by MM. Rilliet and Barthez in their work reviewed in our present Number. To proceed with our analysis.

*Anatomical Lesions.*—The author finds the cases in which the infiltration consisted of serum only to have been comparatively few in number, in three-fourths of those recorded pus existing also. The cases of simple infiltration generally arose in the progress of a general anasarca, as in that supervening upon scarlatina. The folds of the mucous membrane extending from the epiglottis to the arytenoid cartilages are especially the seat of the disease; and in three cases only have the *cordæ vocales* themselves been noted as presenting a certain degree of infiltration. The quantity of fluid effused is sometimes sufficient to produce enormous tumefaction, but few observers have supplied any exact details upon this point. The superior orifice of the larynx then presents two roundish pads, more or less projecting, and, according to their size, offering a greater or less obstruction to the passage of air. They have a tendency to sink down into its aperture, when the larynx is open, and thus, as M. Lisfranc proved by experiments with a bellows, air obtains a far easier egress than ingress, the pads separating in the first case, and approaching each other when air was forced from above downwards. In three cases only has the *mucous mem-*

*brane*, near the infiltration, been found in a healthy condition. In a third of the cases it was red, and in a sixth ulcerated. In one case there was ramollissement, and in another gangrene. In sixteen cases more or less serious lesions of the *cartilages*, especially the cricoid, were found—the infiltration in fact being produced in consequence of the inflammatory action of the mucous membrane, induced by the carious, or other diseased state of the cartilages. The *epiglottis* was infiltrated in four cases, and in eight it was notably thickened. In one case it was ulcerated, and in another covered with a false membrane. In more than half the cases lesions of the *pharynx* were observed, such as coloration, ulcers, or abscess. It thus appears that œdema hardly if ever occurs without being preceded by organic lesions of the adjacent parts.

*Causes.*—The *exciting* causes are therefore the lesions just alluded to; but the *predisposing* ones have for the most part been imperfectly observed by authors. Age seems to exert some influence. Of 38 cases, four only occurred in children less than ten years old; and the greatest number were observed to occur between 18 and 30—the period in which phthisis, the most frequent cause of ulceration of larynx, so often the precursor of œdema, is most prevalent. So, too, this is especially the age for typhoid fever, during the convalescence of which œdema glottidis often occurs. The *sex* has been observed in 40 cases, of which 29 were males and 11 only females. This does not militate against the statement of phthisis being so frequently a predisposing cause; for, although that disease is most prevalent in women, M. Louis has observed that ulcerations of the air-passages are three times more frequent in men than in women. The effects of temperament, constitution, and seasons, have been too seldom observed to allow any conclusions concerning them to be drawn. As to the *prior state of health*, in 4 cases out of 40 only did the disease shew itself as a suffocative angina, the patients being in good health at the time. In ten instances the affection appeared in the course or convalescence of typhoid, or other severe form of fever; and in 12 in the course or convalescence of various other diseases, as pneumonia, scarlatina, erysipelas, &c. &c. In nine cases it followed laryngeal phthisis, in one cancer, and in two syphilis of the larynx. In two cases the state of health was not indicated.

" When an inflammation is developed within, or only even near to, a part of the body where there is abundance of cellular tissue, we soon observe it become more or less engorged with serum or sero-purulent fluid, according to the violence of the inflammation. This is seen to be the case in the subcutaneous cellular tissue in inflammation of the skin; as also in the palpebral cellular tissue, when there is inflammation in the vicinity of the eye, or in the eye itself. This is also seen after a simple section of the prepuce, when the cellular structure often becomes infiltrated in a very notable manner. This effect may be observed in subjects otherwise in good health, but it is much more frequently produced when they have been enfeebled by prior disease, and the blood has become impoverished; or there is a tendency to general œdema. We find here an explanation of what occurs in the larynx when a violent angina affects a healthy subject, and when even a slight angina, having its principal seat in the larynx or pharynx, attacks a subject affected with, or convalescent from, another disease. But to pursue the comparison: if an ulcer is developed with a certain degree of irritation in one of the portions of the body already mentioned, its edges are seen to swell, and the irritation spreading farther and farther, the neighbouring cellular tissue is infiltrated. This effect is remarked in chronic ulcers, when by some cause they become much irritated, as well as in acute ulcers. The same thing is seen passing around an abscess, whether a simple one, or one connected with caries of bone. In studying the facts I have now indicated, one may see, so to speak, demonstrated on the surface of the body, the various phenomena which terminate by producing the serous or sero-purulent infiltration of the larynx, and further, we see the reason of the predilection the œdema assumes for the aryteno-epiglottic folds of mucous membrane, the cellular tissue being here much less compact than elsewhere."

*Symptoms.*—Pain and tenderness in the region of the larynx or pharynx, with or without difficulty of deglutition, has been noticed in nearly all cases. The *cough* and *expectoration* have frequently not been even remarked upon by authors, and are only of a secondary importance. The change of *voice* is a very frequent, if not constant, sign. "It is at first raucous, then marked, then low, becoming in most cases extinguished, or almost so, towards the end of the disease. In one case alone it has been designated as croupal." Although *dyspnœa* is usually a principal symptom, it is in some cases not very marked. In 35 cases out of the 43, however, it has become at times suffocative. As observed by Bayle, the difference of the difficulty in inspiration and expiration is frequently very great, the former being far more noisy and laboured than the latter. In most cases, the inspection of the *fauces* seems to have been neglected ; but in all the 13 in which they were examined lesions of the pharynx, to a greater or less extent, were observed. It is however sometimes difficult to get the mouth sufficiently open. The examination with the finger, too, seems to have been seldom practised, although, in those cases in which it has been done so adroitly, the tumefaction of the glottis has been felt satisfactorily. The digestive organs are not usually much disturbed, but there is great fever, thirst, and restlessness. The countenance exhibits marked change, especially during the paroxysms.

*Progress and Termination.*—The debut of the disease is hardly ever sudden, but once developed, it is often very rapid in its progress. When it results from a chronic lesion of the pharynx, its first announcement may be a suffocative paroyxsm. When it is produced by simple inflammatory action the progress is rapid in proportion to its intensity, and it is then more uniform and less interrupted in its progress. In lesions of the larynx the paroxysms are more distant, and separated by intervals of calm. In some cases the paroxysms are truly dreadful to behold, of frequent occurrence, and long duration. Of the forty cases alluded to only nine were cured. Three only died during the existence of the paroxysm, and seven during a calm interval, in which all seemed going on well. In the other cases, death, although not actually occurring during the paroxysm, did so in the condition of asphyxia, which had become permanent. One perished during the operation of tracheotomy, one ten hours, and another 52 hours, after its performance. The *duration* of the affection is very variable as the circumstances attending it are so different ; and death at periods varying from a few hours in one case, to 26 days in another, has been observed.

*Diagnosis.*—This, which would seem easy enough, has nevertheless in some cases been attended with difficulty. If, with the precursory symptoms, already mentioned, and paroxysms of suffocative dyspnœa, we are able to feel an œdematous swelling at the top of the larynx, by means of the finger passed rapidly into the mouth, this being widely opened, the diagnosis is almost certain; not quite, indeed, for tumours, in the vicinity, have simulated these œdematous swellings, as may collections of matter in the pharynx or œsophagus. Other affections of the larynx itself may render the diagnosis also obscure, as laryngitis terminating in suppuration, the seat of the formation of matter being the posterior walls of the larynx, and generally just above the cricoid cartilage. In one such case only was the pus found in the aryteno-epiglottic folds—the usual seat of œdema. The suffocative paroxysms in this case are much less severe. In *false croup*, we observe that children are almost always the subjects, the symptoms nearly disappear in the intervals of the paroxysms, when the voice becomes almost natural, and no tumefaction is found on the exploration of the larynx. In *croup*, children are also the subjects, and false membranes are usually found in the pharynx. The only pathognomonic sign of œdema is however the presence of the œdematous tumours at the superior aperture of the larynx. Œdema glottidis is sometimes *latent*, and M. Louis reports two or three cases in which the symptoms did not manifest themselves until just prior to death—these patients being already brought into a dying state by the severity of other long-continued disease.

*Prognosis.*—This is of the gravest character, since whatever is done almost all die. The less the lesion which has given rise to the œdema has disorganized the tissues, the more chance there is of a cure being effected, if active means are employed.

"In pronouncing upon the degree of gravity from the symptoms observed, each case must furnish its own elements for decision. In a general manner only we can say that if the strength yet continues, the pulse is regular and strongish, if the features are not much changed, and the face not livid, if the efforts to enable the air to penetrate into the lungs are yet energetic, and if the wheezing or other noise is heard in the larynx with power enough to shew that the air does, although with difficulty, penetrate into the lungs, we may have hopes that the disease will terminate favorably. If, on the other hand, the patient is prostrated; if his features are changed, his lips blue, his eyes haggard, his face cadaveric, as described by Bayle, if he has no longer the power of making the same respiratory efforts he did before, if the inspiratory *sifflement* has lost its energy, without respiration becoming deeper and easier, we must not allow an apparent and deceptive calm to deceive us; for the patient is devoted to a speedy death."

*Treatment.*—Of the whole number of cases collected, nine only were cured. General *bleeding* has been only put into force in seven cases, in some of which it has produced at least temporary benefit. Leeching has been tried in fifteen cases, and in the same number have *blisters* been applied to the neck. In two cases related by the author, *blisters* and *emetics* have been simultaneously employed, and a cure resulted in both. In the one, the œdema came on in the course of a syphilitic laryngitis. Emetics were given, a large blister applied to the neck and two others to the thighs. In the other case the œdema came on in the course of phthisis. Incision, or tearing of the œdematous tumours, and thus lessening their size, has been advocated by some; and the operation of *tracheotomy* by others. Of the 40 cases here reported, this has been practised but nine times, and in three of these life was saved. It must be remembered that the operation has been delayed until the last moment, when asphyxia was imminent, and yet out of the nine cases it has succeeded three times; while in the 31 remaining cases six cures only have resulted. Moreover, in only one case was the disease simple and primary, the others occurring in the course of an acute disease or of chronic laryngitis. To be successful the operation must not be delayed until the last moment, but should be put in force as soon as other methods have been found unsuccessful; but it should not be performed in those patients in whom the original disease is about terminating their career.

### On Hereditary Influence. By M. *Gintrac.*

This paper, upon "the Influence of hereditariness in the production of nervous excitement and the diseases which result from it," occupies nearly 200 pages. The matter it contains might easily have been condensed into a tenth part of that number with advantage, and even then would be found to contain little enough novel or conclusive. We content ourselves with extracting the account of the prevalence of hereditary predisposition as a cause of *Insanity.*

M. Parchappe found, as a result of examining the records of the Lunatic Asylums in France, that of 14,362 admissions, in 1,682 instances, the madness could be referred to hereditary influence. The proportion varied much in different establishments, as from 35 per cent. in M. Esquirol's private asylum to 21, 15, 13, 11, or 10 per cent. in the public asyla. M. Gintrac has furnished additional figures derived from a variety of domestic and foreign establishments, and which, united with those of M. Parchappe, show that hereditary influence has prevailed 3,268 times in 24,012 cases, giving the mean number of 13 per cent. Alluding to the tables quoted, he observes:—

"We may remark the great differences in the results obtained according to the places and observers. In hospitals information is often wanting. It is usually impossible to prove this exactly in patients whose judgment is disordered, and in whom it would be imprudent to direct attention to the causes of their insanity. The relatives have ordinary motives for silence as regards antecedents. It requires a great perseverance, a very strong love for the truth, and a patience beyond all proof, to engage in this description of research and to obtain exact and circumstantial information. Thus it is no wise astonishing that, in hospitals where the importance of this kind of examination has not been appreciated, there has been so low a figure as at Aversa (1 per cent.), while in other establishments it has mounted up to 50 per cent. In the establishment of Esquirol, in the cases of lypemania and of puerperal mania which he has reported, the number of cases of hereditary influence is much more considerable than at Bicêtre or Salpêtrière, because the observations were collected with more care and more details than in those houses where the multitude of the patients rendered their examination less attentive and less complete. It is rather on this account than because of the difference of social position that hereditariness seems seldomer to manifest itself.

"According to Esquirol, insanity is oftener transmissible by the mother than the father. He has been enabled to assure himself of this, during his attendance upon, in the latter years of his life, the children of those who had themselves been his patients at the commencement of his career. * * * Esquirol believes that children, begot prior to the invasion of the disease in the parents, are less subject to it than those who are born afterwards. Whatever may be my respect for this celebrated observer, I cannot admit this entirely, unless in reference only to accidental insanity. For, if the affection is the result of a constitutional predisposition, it little matters whether this is transmitted before or after the fortuitous manifestation of its consequences. It is not the disease itself which parents communicate; for a child is not born mad, but only with the aptitude of becoming so. Now such aptitude may remain latent for a longer or shorter time, by reason of the absence of causes capable of bringing it into action. Thus we may see it traverse one generation without exhibiting itself, and become evident in that which succeeds. A curious example of this may be taken from the observations of Grube. A father, the subject of mental derangement, had sons of distinguished talent who filled public employments with ability. Their children first appeared to possess sound minds, but at 20 years of age they exhibited signs of insanity.

"It is not only individuals manifesting a well-characterised mental alienation who impress upon their posterity a vesanic disposition. Persons of a light, mobile character and capricious disposition, whose ideas are eccentric, tastes depraved, and passions excited, those who are accustomed to debauch and drunkenness, often produce children who, by increase of these unfortunate dispositions, become completely insane."

Of the various forms of *Monomania* some are far more frequently produced by hereditary predisposition than others. This is especially the case with *Suicide,* as has been observed by most writers upon insanity. Of twenty examples collected by M. Falret, five were found hereditarily predisposed, and of 81 collected by M. Cazauvielli, between 1804 and 1833, the same was the case in 22 instances : and frequently, when even suicide has not prevailed, there has been some marked peculiarity of disposition or manners in the predecessors. These observations were made not only in towns but in rural districts, and Zimmerman notices a village in Switzerland, in which there was not a family that had not lost some member by voluntary death.

### THE NATURE AND DEVELOPMENT OF ACCIDENTAL PRODUCTIONS. By *Ch. Baron,* M.D.

This communication is part of an entire work upon the subject to which it relates, presented some time since to the Academy. That portion of it which relates to *Tubercle* has already appeared in the Archives Generales de Médecine, for 1839.

Before considering any of their varieties in detail, the author first states his reasons for considering *all Accidental Productions are of an identical nature and origin.*

1. *The frequent similitude of anormal tissues.*—This is sometimes so great, in some of the intermediate stages of morbid growths, that one is with great difficulty distinguished from another. Thus we may be at a loss to decide whether certain visceral changes should be referred to unsoftened encephaloid, to scirrhous, or to fibrous tissue. The same may often be observed in respect to tubercle and encephaloid in a state of crudity. In some organs the difficulty is greater than in others, and, as M. Cruveilhier observes, nothing is more difficult than the classification of tumours of the brain.

2. *The mutual Transformations which are sometimes operated among some of these productions.*—Thus pus may be converted into a false membrane, cancer into pus, hydatids into tubercle or cancer, cartilage into bone, and tubercle into stony concretion.

3. *Their simultaneous Appearance.*—The author does not agree in the opinion still held by some observers, that different morbid tissues exclude each other. Different morbid products may be observed in different organs, in the same organ, or even in the same tumour. One accidental production may even be developed within the interior of another: *e. g.* tubercles within the substance of false membranes.

4. " *Their Analogy of Structure.*—The masses in all these productions are constituted of small tumours, which we may call initial, and which, in most instances, approach the spherical form. They are formed of various superimposed layers, which seem to converge towards the centre. Frequently the colour and density are not alike throughout the extent of the tumour, the centre being usually the least consistent part. Around accidental tumours is a vascular network, whence branches are sent into their substance in which many are lost, and frequently, in following the vascular ramuscules which penetrate the morbid tumour, we observe they terminate in one of the little granules, the assemblage of which constitutes the mass."

5. *Their Organization and Vitality.*—The majority of these productions are organized, and thus may undergo changes in volume, suppuration, &c. Their vitality, which is restricted, and not alike in all, is due to the vessels which supply them with the material of their nutrition, and maintain their relation to the general circulation.

6. *The Analogy of their Development.*—Most productions are first visible as white or yellowish-white points. Their consistence increases faster than their volume or colouration. This is their period of crudity, which is wanting in some, as pus. Sooner in some than in others the period of softening occurs, commencing in the centre. A more minute inspection shews that, in the earliest stage of their existence, the greater part of these productions are in the fluid state.

7. *The Analogy of Seat.*—Organs well supplied with vessels are those which are liable to accidental productions, and it is in their most vascular portions that these are developed. They are not found, however, in the vicinity of the large, but in that of the small vessels; *e. g.* it is not in the vicinity of the roots of the lungs that the morbid productions of these organs are deposited, while it is the cortical substance of the kidney that is affected with granulations.

8. *Their Generalization.*—The disposition to this is so great, that one may almost say it is more common to find several simultaneous depositions of a given accidental production than to find one only.

9. *The Condition of the surrounding Tissue* is nearly alike in all. During the period of crudity the fibres of this undergo only slight separation and compression, but during the period of softening it offers the characters of chronic inflammation. The morbid product, at the period of its origin, is surrounded by an areolar injection, which has been erroneously considered inflammatory. Sometimes the neighbouring vessels are found to contain more or less coagulated blood.

10. *The Analogy of Symptoms.*—The local symptoms, depending on the obstruction the deposit causes in the organ, are very similar in the various accidental productions, and these cannot be distinguished from each other by their means. The general symptoms, especially manifesting themselves during the period of softening, although they occur at different stages of the disease in different cases, bear a great resemblance to each other, and are especially characterized by the exhaustion of the powers of the frame.

11. *The Ætiology is frequently the same,* as shewn in the effects of hereditary influence, inflammation, congestion, contusions, &c.

12. *The Chemical Composition* is remarkably similar—the differences being almost wholly due to the varying proportion of the same elements.

"The reasons which I have now assigned seem to me to sufficiently prove the identity of the origin and nature of accidental productions. What I wish now to show is *the identity of non-analogous morbid productions and of analogous morbid productions.* The propositions above enounced nearly equally apply to these two classes of productions, and we may refer especially to the frequent simultaneous existence of analogous and non-analogous degenerescences. Thus, one often meets with, in the same subject, and often in the same tumour, encephaloid matter, tubercular and osseous, scirrhous and fibrous tissue. Considering then these analogous and non-analogous productions identical, it results that the origin of morbid tissue is the same as that of the normal tissues. It is indeed impossible to deny that accidental cellular and tendinous tissue is identical with normal cellular and tendinous tissue, and as we admit the analogy of the analogous and non-analogous morbid products, we must admit the community of origin of normal and anormal tissue. Professor Bouillaud observes, 'the laws which preside over the formation and development of the anormal tissues are but modifications of those to which the formation and development of normal tissues are submitted.' This analogy is further proved by the vascularity and evident organization of the accidental productions, their participation in the general modifications of the organism, and their vitality."

Trousseau and Leblanc, and others, have referred the origin of these productions to a *sub-inflammation;* but no inflammatory process is usually set up around them unless and until softening occurs, and, if it existed, would produce very different results, accordingly as these productions were situated in one organ or another. The opinion of Cruveilhier, that they are due to a *phlebitis*, because coagula are sometimes found in adjoining vessels, is not more tenable.

These anormal tissues are generated from the *blood*, and therefore an afflux of this fluid, or even in some cases inflammatory action, is favorable to their production. It is difficult to go beyond mere hypothesis in stating how these changes in the fluid are affected. However, the development of certain products from effused and coagulated blood has been observed. "We see the blood arrested in its vessels or effused into the tissues, and the resulting coagula themselves induce a true secretion, a separation of the elements necessary for the formation of the morbid production. Certain elements of the blood disappear by absorption, and others are subjected to successive transformations, whence result the anormal products." The names of Abernethy, Forget, Andral, and Piorry, are cited n proof of this generation of anormal productions in coagula.

*Encephaloid Matter.*—After describing several autopsies, in which the gradual conversion of a coagulum into encephaloid matter was observed, the author thus sums up his observations:—

"In a great number of cases a coagulum is deposited in the organic tissue, or is yet contained in the vessels. The serum is first absorbed, and the colouring matter is removed, the concretion becoming fibrinous, yellowish, then whitish, and augmenting more and more in consistency. The matter then becomes smooth and lardaceous like dense cerebral matter, a slight rose colour pervading it. The degeneration is usually more advanced as we approach its centre, so that sometimes the encephaloid matter is completely formed and even softened at the centre, while the circumference is yet constituted of fibrinous blood, or of blackish red blood, the consistence of gooseberry jelly. At a later period, the external layer of the production may become converted into an enveloping cyst, or may act as a partition in a mass when many tumours are conjoined. The cyst is often however formed of the cellular tissue of the organ in which the tumour is deposited.

"The mass increases by the addition of new material which the numerous vessels that traverse it supply, by the conversion into encephaloid of the effusions of blood which occur in its various portions, especially towards its centre, and after by the union of many neighbouring tumours."

After offering a similar explanation of the origin of *colloid* or *gelatinous cancer*, the author next presents several observations upon

*Hydatids of the Lungs and Pulmonary Apoplexy.*—The blood effused into the texture of the lung, constituting pulmonary apoplexy, when not absorbed, may undergo great change. The most excentric layer of the effusion may have undergone absorption, leaving a yellowish intermediate layer containing blood in its cavity. Sometimes, as this fluid internal portion of the blood disappears, this yellow envelope contracts and remains a persistent cicatrix. In other cases, the fluid is removed from the centre by communications with the bronchi, or by the interior of the envelope taking on the secretory action, and a hydatid cyst is left, which becomes lined with a smooth polished membrane, and filled with a serous fluid.

"I add, as a proof in support of this mode of the origin of hydatids, that a common cause of their development is a fall or shock, such as may produce a commotion and effusion of blood in the organ which contains them. Further, we often see morbid tumours constituted at once of serous sacs and of other accidental productions, due generally, as I believe I have shown, to the transformation of the effused blood. This simultaneousness of development in the same parts of the economy seems to prove that the acephalocysts and other accidental productions have a common origin. Lastly, as M. Berard states, it is in the spongy portion of the long, and the diploe of the flat, bones, *i. e.* in the most vascular portions, that hydatids ordinarily develop themselves. So too, within the cranium, it is chiefly near the pia-mater, the vascular network enveloping the brain, that hydatids arise, and especially in the fissures, in which are lodged the principal arterial branches.

" To those who may feel surprized that the most excentric layers of a coagulum should be connected with an hydatid cyst, non-adherent to the cavity containing it, I may answer that the walls of the acephalocyst possess all the properties of fibrine and albumen, and that, since the work of Legroux on Sanguine Concretions, it is impossible to doubt that a coagulum may be converted into a cyst; and that M. Bouillaud, among many other authors, cites numerous enough examples of coagula being so converted into simple or compound cysts. We must not regard the transformation of the pulmonary apoplectic deposits as a fiction, drawn from a rational induction, but as a fact resulting from observation; for, as cerebral apoplectic *foyers* are frequently converted into serous cysts, the occurrence of the same thing in the lung can hardly be disputed."

*Melanosis.*—After citing the observations of several authors, to prove that melanosis is produced by a transformation of the coagulum of the blood, the author observes :—

" The chemical composition of melanosis has thrown much light on its nature. All chemists who have analyzed this matter have remarked its great analogy to the blood. It is seen, by the analysis I have given above, that fibrine, albumen, oxide of iron, and many salts, are found in it in notable proportions, and very similar to those which exist in the blood. But there is a principle to which we must direct some attention, namely *carbon*, which is found so abundantly in melanosis as to constitute one-third of its substance. As this principle only exists in small proportion in the blood, a supplementary quantity must be formed to constitute melanosis. The proportion of carbon is probably not the same in all melanoses, being feebler in those which are soft and reddish brown, than in those which are black, dry, and dense. It is easy to prove that melanosis is constituted by the combination of most of the elements of the blood with a considerable quantity of carbon, but it is not so to discover the origin and mode of formation of this carbon. According to MM. Barruel, Foy, Trousseau and Leblanc, the cruor is the only element of the blood whose change can furnish it. This theory of its origin is a rational one. Nevertheless, I believe carbon may also be derived from other sources. Thus, in the lungs, a portion may be derived from the chemical act of respiration. A part retained in the lungs mixes with the blood, whose coagulation it produces, and from its mixture with this coagulated blood, results the melanosis. The carbon of pulmonary melanosis may also be in part produced by the combustion of bodies employed in artificial illumination."

*On the Black Pulmonary Matter.*—The author in concluding his paper details the usual appearances which the deposition of the black pulmonary matter presents. It is found at the surface of the lungs, either in the form of mere points, lines, or superficial layers. On examining the interior of the organ, points and lines of black substance are found chiefly in the interlobular tissue and augmenting in size as they approach the roots of the lungs. Most of these become continuous with small vessels which have exactly the appearance they would have if injected with some solidified black matter. In the bronchial glands at first a black point is observed, then layers of black matter, and at last a sort of general infiltration, which hardly allows the normal tissue to be distinguished. Sometimes it is deposited in amorphous masses.

Dr. Baron considers this black pulmonary matter to be primarily deposited within the bronchial veins; and that its presence in ripe and advanced age is one of the causes to which the diminution of the respiratory powers may be attributed. He regards it as coagulated blood conjoined with carbon. The coagulation may be produced by the presence of carbon, which may be derived from the chemical act which constitutes hæmatosis, and from the combustion necessary for artificial illumination.

### On the Acute Delirium observed in Establishments for the Insane. By *Brierre de Boismont*, M.D.

Dr. De Boismont, having related several cases of this affection at considerable length, next proceeds to give a general view of its principal characteristics.

*Symptoms.*—The delirium in these cases does not usually show itself suddenly, but the patient, for some time prior to its occurrence, manifests a change of character, habits, and tastes. Naturally of a placid disposition, he becomes morose, quarrelsome, prodigal, covetous, &c. Persons who have manifested great indifference to religion may become minutely attentive to its observances, while others, to whom such was habitual, now neglect them. Some persons exhibit great volubility of speech and others an incoherency of ideas. Such precursory signs having existed for days, weeks, months, or even years, the delirium at last exhibits itself. The ideas now seem to come and go without order or relation to external objects, and often succeed each other with a rapidity it is impossible to follow. The nearest relatives cannot excite the attention of the patient, or obtain replies to the most urgent questions. The paroxysms are often of the most violent character, the delirium frequently, however, revealing some predominant idea, and that of a desponding character, especially in those of a religious turn. The violence of the delirium is sometimes interrupted by more or less completely lucid intervals. There is a peculiar mistrustful and sinister expression of countenance generally observed. There are two well-marked stages of the affection, that of excitement and that of succeeding exhaustion. The period of their respective duration is very varying, the debility sometimes coming on in a few hours and at others not until just before death. At the commencement of the affection the patient is in constant motion, often displaying immense muscular power, and generally requires, for the preservation of those around him, to be secured. Frequently, various of the muscles are powerfully convulsed, so as sometimes to amount to complete epilepsy. Grating of the teeth and spasm of the œsophagus are especially observed. Some patients, however, remain motionless from the commencement, as if a prey to the deepest melancholy.

Paralysis, either general or local, which is one of the symptoms of meningitis and encephalitis, is not found, or very rarely so, in acute delirium.

At an early stage of the affection the moans and cries of these patients are so loud and penetrating that it becomes necessary to place them at the greatest distance from other patients. Their shrieks usually diminish as the malady advances, but not always. Some of the patients are tormented with hallucinations and illusions, but the most characteristic symptom in this affection is a *hydrophobia*, or aversion to liquids. Sometimes this arises at an early, sometimes at a later stage, but the savage energy with which attempts at giving drink are resisted can hardly be conceived.

"This refusal of drinks may continue for several days, and it is common to observe patients who have drank nothing for 24, 36, or 48 hours, and even for three, four, five, or six days. We had under our care a lady who, for twenty days, took no liquid but by means of an œsophagus-tube. This symptom offers more or less marked remissions. Patients who had refused all liquids, consent to drink at a time when they were least expected to do so. In the greater number of cases the hydrophobia diminishes with the progress of the delirium. The symptom may, however, persist during the whole course of the disease, being generally remittent and sometimes intermittent. A protestant minister had six attacks of acute delirium, with refusal of drinks each time. Among facts of this kind we may cite the following:—a young lady drank the first day with avidity; the second day there was less thirst, the third day, after some hesitation, she obstinately refused to drink at all. During three days she took *tisanes*, or rejected them with rage; the seventh day she drank all that came before her; the eighth day nothing could persuade her to take any fluid. She gnashed her teeth in fury when a glass was presented, and buried herself under the bed-clothes. This obstinate refusal continued during the tenth, eleventh and twelfth day, when she died. When moribund and speechless, presenting the *facies hippocratica*, she closed her lips and turned away from the glass which was presented to her."

In most cases there is, indeed, the same repugnance for *food*, so that whatever nourishment is taken has to be administered by a tube. Those who die are reduced to the extremest marasmus, which often comes on with great rapidity. Some patients eat with avidity, and others will only do so at prolonged intervals. There is always *fever* present. The pulse is usually from 100 to 120, full and hard, but at other times small and soft. In the course of the disease it loses power and gains in frequency. Very frequently neither bleeding or abstinence seem to exert any effect upon its acceleration. The tongue is almost always very rough, but not red, and in a few days becomes white, but sometimes dry and brown. Constipation is almost always present, and the cutaneous transpiration is generally suppressed. A penetrating and abominably fætid odour is exhaled from the skin. Sleep is almost entirely absent during the early stages of the disease, and resembles stupor when it occurs later. The recurrence of natural sleep is a very favorable indication.

*Pathological Anatomy.*—After examining in detail the results of seven autopsies, M. B. thus concludes:—

"To recapitulate; acute delirium may leave no traces of anatomical change. (Such was the case in two out of the seven cases.) In some cases there is found a simple injection of the membranes and of the brain, while, at other times, this is accompanied by the lesions of chronic meningitis or general paralysis. There is also sometimes found some of the appearances of ramollissement, of meningitis, of encephalitis, or of meningo-cephalitis. We conclude from these facts that acute delirium, like insanity, has no lesion which is peculiar to it. The modifications in texture, colour, and consistence, which sometimes accompany it, nowise differ from the alterations recorded by observers in mania, dementia, and general paralysis. These pathological conditions being themselves but the results of congestion, of meningitis, and of encephalitis, it seems at first natural to conclude that acute delirium and mental alienation are but inflammations of the membranes and cerebral substance. This conclusion, inadmissible as respects insanity, has no better foundation in acute delirium. All that can be reasonably stated is, that these different morbid lesions are but complications of the disease which now occupies our attention."

*Causes.*—These are not always to be ascertained, since the patients are not in a condition to throw any light upon the subject, and when the disease comes on suddenly, it often seems to be unconnected with any antecedents. In the majority of cases, however, careful inquiry shows that "the affection has been preceded, favoured, or produced by cerebral diseases, paroxysms of insanity, or hereditary influence." Of 19 examples here cited, in seven cases the delirium appeared suddenly, and in 12 there were premonitory symptoms. The exciting causes are generally the same as those of insanity. Of the 19 cases, 11 were men and 8 women; 14 married and 5 single persons. The ages of the 19 ranged between 17 and 66, the greater number of cases occurring between 40 and 50.

" *The Diagnosis* of acute delirium presents great difficulties. Almost all authors, in fact, make it a pathognomic sign of meningitis, of encephalitis, and of meningo-cephalitis. Can we feel astonished at the vagueness of the information concerning this disease when we recollect that, a few years ago, the general paralysis of the insane was unknown to physicians, and that, even now, it is the source of frequent errors? What constitutes the difficulty of the diagnosis of acute delirium is, that it offers numerous points of contact with inflammation of the brain and its membranes, varieties of insanity, certain ramollissements, low fevers, insidious eruptive fevers, violent visceral phlegmasiæ with re-action on the brain, and many neuroses. * * * The opinions of ancient and modern authors present numerous differences as regards the symptoms, the nature, and the identity of the disease; for, while some attribute it to a lesion of the membranes and of the brain, others describe it as a special disease, destitute of known anatomical characters, and consequently belonging to the category of nervous affections."

Of all affections *meningitis* is that most likely to be confounded with it, but yet there are important marks of difference. In acute delirium there is no headache, a constant symptom in meningitis, coma never occurs at an early stage, nor is there ever seen contraction of one side, paralysis, nausea, or vomiting, all which are common in inflamed membrane. In the former disease the mental disorder bears relation to the character of the individual or the cause of the delirium, while, in the latter, it consists of unconnected cries and vociferations, or resembles mumblings and ramblings of a drunken man. In meningitis there is not the utter repugnance for food and especially drinks, there is not the excessive agitation of the body, or sinister expression of the eye. *Congestion* differs by the suddenness of the access of the symptoms, and the rapidity of the progress and termination of the affection. In *acute insanity* there is generally not so much fever and a more regular circulation; sensibility and response to external sensations are more complete; there is less concentration of thought, and more disposition to hallucination and illusion, and no convulsive movements; the disease does not last long, but terminates by cure, chronic mania, dementia, with or without general paralysis, or by death in a state of acute delirium or acute mania.

The *nervous delirium* of Dupuytren, occurring in persons who have met with accidents, sustained operations, &c., is characterized by a singular confusion of ideas of persons, places, and things. Sleepless, the patients become the victims of some dominant idea. They remain in a state of continual jactitation, and with a flushed face, and brilliant, injected, eye; they give vent, with an extraordinary velocity, to menaces and frightful vociferations. Their insensibility is such that they can employ their broken limbs, and the pulse is quiet. In four or five days the disease is terminated by death or cure, usually the latter. The rapidity with which this delirium is relieved by laudanum-glysters distinguishes it easily from the other form. *Delirium Tremens*, also, sometimes may be mistaken for this disease. The patient is continually gesticulating and speaking, engaging in imaginary labours, and in conversations concerning his ordinary occupations and continually recurring obstacles. The perspiration pours down in consequence of this morbid activity. Frequently there is little or no fever. The tremor, whence the disease derives its name, may be absent, and the diagnosis of this species of delirium is often chiefly derived from a knowledge of the habits of life of the patient, and observation of the nature of his hallucinations. Opium, and in some cases mere rest, effect a cure. The onset of *typhus* and other *fevers* may be attended by a delirium, difficult to distinguish at first from simple acute delirium; but, in the progress of the febrile affection, the manifestation of other symptoms comes to our aid.

" *Nature of the Disease.*—The characters which separate it from meningitis and meningo-cephalitis are well-marked. If sometimes they are distinguished with difficulty, it is because complications have occurred, and the diseases have become confounded. In its simple state, acute delirium is a nervous affection, which doubtless corresponds with a modification of the cerebral organ; but such modification is unknown to us. It is with regard to the proximate cause of acute delirium as with those of the delirium after operations, of drunkards, from the ingestion of narcotics, and of the calenture or delirium of sailors—to the present time they have eluded all researches. Congestion is but an accessary of the delirium, which may besides be absent. Supposing even that the inflammatory element always accompanied this delirium, which is not the case, it would not constitute it, any more than it constitutes syphilis, cancer, the exanthemata, and many other pathological conditions.

" It may be asked whether acute delirium is insanity or a variety of that disease. The acuteness of the affection, the symptoms, and the general physiognomy, are so many replies in the negative; but if, on the other hand, we study the etiology, the terminations, and some of the symptoms, we shall be obliged to acknowledge that acute delirium approaches some of the acute forms of insanity. If this delirium be not insanity in the generality of cases, why isolate the patients, and place them in lunatic asylums? The reasons for this proceeding seem peremptory. Most of the subjects of acute delirium are noisy and agitated; they vociferate and howl, so that their residence in private houses is impossible. Many of them escape undressed, throw themselves out of windows, kill themselves, fight with those who are about them, and frighten the assistants. Moreover, at its commencement, the disease is not easily distinguished from a paroxysm of insanity. The necessary means are much more easily administered in such institutions than at home. The mere sight of the establishment has sufficed to recall the reason of many of these persons, and the best answer to objections to them is the fact that many individuals have rapidly recovered by the means therein employed."

*Duration and Prognosis.*—The duration is usually very short, from two to eighteen days, and the longer it continues the greater is the danger. When the delirium is in its simple form the prognosis need not in general be a grave one. A young lady observed a rigorous fast for twelve days and was yet perfectly cured. Symptomatic delirium also, such as that arising from recession of eruptions, rheumatism, &c. usually is cured by appropriate remedies. The prognosis is very bad when the hydrophobic symptoms exist, and especially if they persist. If the agitation, cries, and convulsive movements continue five or six days the termination is almost always fatal. A very fatal sign is the exudation of a small quantity of muco-purulent fluid from the angle of the eye. The remission of symptoms prior to coma also augurs badly. We must not be deceived by lucid intervals, which are sometimes of long duration; but when they are attended with mobility and brilliancy of the eye, restlessness of the body, hot skin, and a quick pulse, a new paroxysm is imminent.

*Treatment.*—As in insanity so in this affection *bleeding* must not be looked upon as a sovereign remedy: but it is more useful in this than in that affection, and at the commencement of the attack should unhesitatingly be employed, judging of the extent required by the pulse. In almost every case a careful exploratory bleeding is at least admissible; and the general condition of the patient must guide us as to repetition. We must not, however, be misled by the mere violence of symptoms; for if we continue to bleed or to subdue these, connected as they often are with a chronic meningitis, we only hasten on dementia or death, and in any case retard convalescence. Bleeding from the *foot* has proved more useful than from the arm, especially in strong plethoric persons, liable to congestions, hæmorrhoids, and suppressed menses. In most cases the arm is however to be preferred. Local bleeding by *leeches* is an useful adjunct. They may be applied at the side of the neck, the base of the cranium, or on the shorn scalp, in the course of the sagittal suture. In suppressed menstruation, the interior of the thighs, and in hæmorrhoids, the anus, are eligible sites. Cupping the nape or behind the ears is useful; but in most cases all these three means of depletion should be simultaneously employed.

*Baths* are one of our most valuable means, especially in the hydrophobic form of the disease; but to be of service they must be continued for five, six, eight, ten or twelve hours; for it is not until several hours have been passed in the bath that the agitation becomes allayed. The desire of the patient to be removed must not be complied with, and the persons around them must tell them they have not the power of assenting to it; for the insane are as credulous as children. The temperature should be from 75° to 85°.

*Cold affusion*, or rather irrigation, is inseparable from the baths. A watering-pot held the height of a man may be employed, and the affusion, which has a very calming effect, should be repeated several times during the bath. Ice or wet cloths must also be applied to the head; but the *douche* does more harm than good. Pediluvia and sitting baths are of use in suppressed hæmorrhoids and menses.

*Emetico-purgatives*, as antimony and calomel, when they produce purging and vomiting, are very useful; but frequently castor-oil has succeeded in producing abundant stools when far more violent purges have been tried in vain. A weak broth best masks its flavour, when the patients will take it.

"The effect of *purgatives* merits the attention of practitioners. Patients delirious, unable to comprehend any question, have been no sooner purged than they have in part recovered their understanding, and been enabled to furnish details as to their condition. Others plunged in a deep torpor, in a kind of stupor, awake, and seem to be re-vivified. In some patients whose countenance has exhibited the peculiar sinister appearance, purgatives, like bleeding, have caused the disappearance of this symptom. We cannot too often repeat that evacuants have proved of the highest utility in the treatment of this delirium: more than once their action has been instantaneous, and under some circumstances we have been obliged to prescribe them alone, the other remedies suspending their good effects. The use of purgatives should be ordered early, for the progress of the disease is so rapidly fatal, that art has need of every resource. They should be given the next day after bleeding when this shall have been necessary, and in the hydrophobic form of the disease the two remedies should be employed simultaneously. We must repeat the use of these therapeutical agents, until a marked amelioration is obtained, at least unless there are counter-indications. They must be varied, replacing some by others, according to the idiosyncracy of the subject. Purgative enemata, when they can be administered (which the restlessness of the patient often renders a matter of difficulty) speedily relieve the commonly attendant constipation."

*Revulsives* are particularly useful as the stage of agitation is subsiding and that of prostration is commencing. At a later stage they are of less and even of no use. Blisters are especially useful when there has been some prior exanthematous or rheumatic disease, as also when there exists any erratic affection. They exert most effect applied to the lower extremities. Setons are improper at the early excited stage, but are very useful when the patient does not seem to enter into convalescence, but becomes apathetic, dull, and melancholy.

*Narcotics*, as laudanum, are more successful in tranquillizing inordinate excitement when given in the form of enema than when given by the mouth. In the stage of prostration, *tonics* and *stimuli*, of which latter camphor and musk are favorably spoken of, are required. When the disease puts on an intermittent form *quinine* is very useful. The administration of *food* and *drink* is often a matter of great difficulty and only to be accomplished by the stomach-pump, or in some cases by glysters.

*Restraint.*—" Should these patients be confined to their beds, or be allowed their freedom, the necessary precautions being taken? When they lie down quietly and will not remain up, it would be hurtful to oblige them to rise and to walk; but when they will not remain in any one place and seem tormented with the desire of moving about, the question is a more difficult one. Have the coercive means employed to restrain them the effect of increasing their fury and thus aggravating their condition? More than once, while observing the violence of these patients, the efforts they make to unloosen themselves, and the rage the powerlessness of these efforts cause, we have asked ourselves whether it would not be better to abandon them to themselves. On the other hand, it appears singular to allow the wandering about of individuals affected with fever, exhaling a fetid odour, and passing several days without eating, drinking, or sleeping. Nevertheless here is a case worthy of attention. M. B. was the subject of a most furious delirium; he recognized no one, sang, cried, vociferated, continually, and never ceased walking night or day. His pulse was 120, skin hot, breath fetid, mouth and lips dry, tongue covered with a brownish yellow paste. Having placed a strait-waistcoat upon him, I allowed him his liberty in a court-yard. He walked backwards and forwards, and leaped with prodigious activity. For ten days it was impossible to make him drink; he became reduced to the last stage of marasmus, and a purulent discharge oozed from his eyes. He could hardly at last sustain himself, and was obliged to sit down every minute. Towards the twelfth day he asked for a little water, and afterwards partook of some fruit. Towards the twentieth day he became convalescent. During all this time he never ceased walking, except when fatigue obliged him to lie down upon his bed. M. C. attacked with furious acute delirium, abstinence from drink, and sleeplessness, was left to himself. During five days his activity was prodigious, but on the morning of the sixth day he became pale and died in two hours. Mrs. —— was affected with fever, flushed face and hot skin, the excretions being suppressed and the expression of countenance sinister. For twelve days she had not slept, and her husband stated she was continually walking, so that it was impossible even to undress her. Privation of food and drink had endured for eight days. She remained four more days in my establishment without taking any thing, pacing her chamber, night and day. On the thirteenth day of her fast she entered the ladies' refectory, and on drink being offered her she took it. With a little precaution as to diet she soon became convalescent. These facts seem to show that there are circumstances under which it is better to leave the patient at liberty, although he may be the subject of fever and thirst, and self-condemned to fasting. So true it is that Nature guides us to health by such different routes."

### On Interstitial Extra-uterine Pregnancy. By Dr. *Payen.*

A woman, pregnant with her second child, having enjoyed excellent health, and led an active life, was seized one evening with violent pain in the hypogastrium and afterwards with syncope. She expired in a few hours. At the autopsy a large quantity of blood was found in the abdomen. The uterus was found to be as voluminous as at the second or third month, and at its upper part a prominence was observed, which, from the diaphanous state of its walls, admitted an embryo floating in its amnial liquid to be seen. The uterine cavity was of a size equal to the development of the organ, but contained no ovum. It was lined throughout its whole extent by a thick conscrescible substance, forming a kind of imperfectly-organized mucous membrane, which almost entirely filled it.

No hæmorrhage had occurred into the cavity. Above this cavity was another, evidently formed from the distension of the substance of the uterus, and situated at its superior and left portion, near the uterine extremity of the Fallopian Tube. An accidental incision prevented the ascertaining whether the tube terminated in this cavity; but this is highly probable, since its uterine extremity was not discernible on the left side, though distinct enough upon the right. The partition separating the two cavities was very thin, but no communication between them could be traced. The fœtus and its placenta attached to the upper portion were found entire in this interstitial cavity, the whole tumour containing it being equal to about a duck's egg in size. The walls of the uterus became gradually thinner as they covered it, until at last, at the upper part, they were transparent. The author states the hæmorrhage resulted from a rupture of the uterus, but he does not indicate in what part of the organ this occurred. Some inexpert *experts*, who, in consequence of the suddenness of the woman's death, and the suspicion of her friends, were charged with the examination of the body, came to the conclusion that these appearances and her death resulted from an abortion produced by the employment of some pointed instrument. The author's refutation of this opinion delivered to the officers of justice, we need not detail.

### ON THE SECRETION OF URINE IN CASES OF POISONING BY ARSENIC. By *O. De Lafond.*

The experiments of M. Orfila, leading to the conclusion that the urinary secretion is continued during acute poisoning by arsenic, and those of MM. Flaudin and Danger, that it is suspended, the author, who is the "Professor of Legal Medicine in the Veterinary School of Alfort," undertook farther experiments upon horses and dogs, in order to endeavour to decide the question; which is not only an important one as regards the affording legal proof of poisoning having been effected, but also in reference to the administration of diuretics as a remedial measure, as recommended by Orfila. The particulars of twelve of these experiments are given in full detail, as is the account of the various preliminary precautions which were taken; but for these we must refer to the volume itself, having only space for some of the conclusions.

"1. The duration of the poisoning in horses has been one hour, one hour and a half, eight hours, 21, 29 hours, and never more than 51. In dogs it has been five hours, eight hours, but not beyond twelve hours. 2. The symptoms observed during life and the lesions after death have completely demonstrated acute or sub-acute poisoning. In some of the animals the inflammation of the mucous membrane was so violent, that in an hour it produced the formation of several metres of cylindrical false membranes. 3. In none of the animals has the secretion been suppressed during the poisoning. 4. The mean proportion of urine secreted in an hour by animals in good health, compared to that secreted during the same time by animals of the same species which have been poisoned, is for the horse : : 347 : 100, and for the dog : : 24 : 4; a proportion which shows that the secretion is not suppressed, but very notably diminished. 5. The urine does not begin to yield the poison until very evident symptoms of poisoning show that the arsenic has been absorbed, and is accumulating in the blood.

Nevertheless the time which has elapsed between the administration of the poison and its detection in the urine has never been less than one, and never more than seven hours."

### On Penetrating Wounds of the Abdomen with Extrusion of the Omentum. By *Hippolyte Larrey*.

Jean Petit, æt. 22, and of strong constitution, was stabbed in the left flank with a knife. He walked to the hospital without losing much blood. On examination, the omentum was found to have issued from a penetrating wound, and was not attempted to be returned by the house-surgeon on duty. When M. Larrey saw him next day he found a wound of about six millimetres in length, situated just above the crest of the ilium, and projecting from it a lump of omentum of about the size of the two thumbs conjoined, recognizable by its fatty texture, and like most traumatic herniæ, deprived of peritoneum. The tumour was swollen and tender but nothing like gangrenous inflammation, which sometimes rapidly supervenes, had set in. The patient's general condition was satisfactory, and no sign of internal hæmorrhage existed. A few attempts at reduction were made but not persevered in, owing to the tenderness and strangulated state of the omentum. It was covered with oiled lint and a poultice; the most absolute repose was insisted upon, a bleeding practised, and complete abstinence enjoined.

In the first day or two the omentum became much inflamed and engorged, but this state was much relieved by applying leeches in its vicinity and the use of baths. On the 4th day a healthy suppurating surface became established, and some time after the danger of further strangulation was lessened by the formation of a small ulceration at the internal angle of the wound. Towards the tenth day the omentum, which had continued firm and tender, and was freely suppurating, began to diminish in size and to slightly retract, and from this time the gradual lessening and disappearance of the swelling went on, these being aided after a-while by the application of nitrate of silver to the irritable surface, and the compression of a bandage. By the thirty-sixth day the tumour was reduced to the level of the skin, and in ten days more cicatrization nearly completed, the wound having contracted to a small aperture, which continued to the last very sensitive to the touch.

The report concludes by some general observations as to what is the best mode of treatment of cases of protrusion of the omentum. Immediate *reduction* by the taxis is the most natural and simple plan to adopt, when there are no counter-indications. Should the reduction be facilitated by an immediate or consecutive enlargement of the wound? When the wound is near the stomach or liver, and violent symptoms arise from the dragging made upon these viscera, the too narrow wound should be enlarged. But this is not to be done for temporary hiccough, vomiting, &c. which may be but sympathetic. It is not indispensible therefore, in all cases, and it exposes the patient to the risk of an issue of a larger portion of the omentum, as well as all the inconveniences of a too large cicatrix. The old surgeons almost always detached the omentum by means of a

*ligature*, but Ambrose Paré pointed out its dangers, which were demonstrated also fully in the experiments of Pipelet and Louis. *Excision* is only practised at the present day for the removal of any portion that may have become gangrenous.

"To wait, and only employ preventive or palliative means, seems to be the wisest and most rational conduct, whenever the traumatic hernia does not present any grave complication. Pipelet and Louis in multiplied experiments, which have so well exhibited the inconveniences of the ligature, have also proved that, if the omentum be left hanging out, without excision or ligature, no ill effects result after its reduction, even when it has been handled roughly. * * * * * More than this, if gangrene has passed the aperture, and proceeded for an inappreciable depth towards the viscera, nothing better than a simple palliative treatment can be enforced. As to the old practice of drawing a portion of the mortified omentum without the wound, to discover the healthy part, it is a vicious practice which has been generally disapproved of since the time of Pipelet.

"A few words upon the secondary treatment which is essential for securing the success of the expectant method. I hope I may here be allowed to invoke the great practical authority of my father, who seems to me to have alone well demonstrated the consecutive reduction under the influence of this method, in favour of which he has formally declared himself. What should be done then? Envelop the omentum in lint soaked in oil, or better, in a compress spread with ointment, to protect it from contact with the air, and from adhering to the circumference of the wound. Some fine lint, one or two compresses, and a bandage to support the whole without constriction, completes the apparatus. If the omentum is swollen, red, and painful, a cataplasm between two pieces of linen, and local bleeding at the margin of the wound are required, cupping being more useful in this case than leeches."

The position of the patient should be flexed and easy, and if peritonitis, colic, &c. supervene, the appropriate treatment must be employed. The local inflammatory symptoms at first augment, and the omentum becomes more tumefied: but, after a while, suppuration, which prepares the parts for their future reduction, occurs. When this diminishes, the exposed part lessens in size, tenderness, and redness, and is gradually retracted towards the cavity of the abdomen. This latter stage is expedited by the application of caustics and the use of a bandage. A firm cicatrix opposes the production of any consecutive hernia in this site.

The desire to present our readers with a full view of this volume in one article, has deprived us of the space we might otherwise have employed in remarking upon its various contents.

